# Prophylaxis and Treatment of Typhoid Fever

**Published:** 1908-06

**Authors:** John B. Woodville

**Affiliations:** Fayette, W. Va.


### PROPHYLAXIS AND TREATMENT OF TYPHOID FEVER.*

BY JOHN B. WOODVILLE, M. D., FAYETTE, W. VA.

When we consider the wide geographical distribution of typhoid fever, its endemic prevalence in many localities and frequent epidemic outbreaks with destruction of life and damage to business in the communities affected, the importance of the problem of prophylaxis is forced upon us, and we realize the truth of the old saying, "an ounce of prevention is worth a pound of cure."

With the rapid advances in medical science of recent years, especially along the line of bacteriology, the question of prevention of some of the most formidable diseases is no longer in its incipiency but is an accomplished fact. Yellow fever and malaria, the scourges of the South, are rapidly being eradicated with the destruction of the mosquito. But the causative agent of typhoid fever, the bacillus of Eberth, not being dependent upon the body of a host for existence, but being capable of living and some-

*Read before the Layette County (W. Va.) Medical Association, May 5, 1908.

times multiplying in earth, in cesspools, in drinking water, also on fruit and vegetables and other articles of human diet, even in ice, its destruction *en masse* is impossible and the question of prophylaxis narrows down to the tracing of the source of infection in a given case and the prevention of its spread in the community. As we are not likely to become aware of the presence of the typhoid bacillus prior to the development of a case in our midst, our duties towards prophylaxis begin upon the diagnosis of the first case in the neighborhood. If the bacillus can be found in the water supply, of course the use of that water must be prohibited; or if it is the only source of supply for the family or neighborhood, the water must be boiled before being used. Not only is this precaution necessary for the drinking water, but all water used for household purposes whether for bathing, washing dishes, floors, windows, etc., if procured from an infected source must first be boiled.

Of the utmost importance is the immediate removal from the room and thorough disinfection of all discharges from the patient —feces, urine and sputum. The best and safest means of disposing of these excreta is by burning, but when this is impossible, various chemical antiseptics are at our disposal. Chemical disinfection of typhoid excreta is surrounded by difficulties and is uncertain—the best antiseptics requiring from one to twenty-four hours of continuous contact with the infective material in order to render it free from danger. The best agents for this purpose mentioned in the order of their efficiency, are lime, bichloride of mercury and carbolic acid. Formaldehyde is also used but it is doubtful whether it has any advantages over lime as a disinfectant for typhoid excreta.

Whatever agent is used, it must be thoroughly mixed with the stools by stirring with a stick, breaking up all solid lumps, the stick being immediately burned.

Lime in the form of solution of the chloride when mixed with typhoid discharges requires one hour to render them innocuous. Bichloride of mercury requires six hours and carbolic acid twenty-four. Chloride of lime being the most effective and the cheapest will likely remain the disinfectant of choice with the country practitioner.

Six ounces of the powdered chloride to a gallon of water makes the proper strength. The discharges after being disinfected unless burned, should be thrown into trenches four feet deep and covered with earth.

The bed pan and all vessels and dishes used by the patient should be washed in boiling water. The person of the patient must receive strict attention as regards cleanliness and the clothing and bed linen should be boiled before being sent to the laundry or given to the washer woman. This precaution should be always observed since it is a well known fact that washer women may and sometimes do, contract typhoid fever as a result of washing infected clothing; and being careless in the disposal of the wash-water, might throw it where it would drain into a spring or well and thereby cause an epidemic.

Physician and nurse must wash the hands with hot water and soap, then in 1-1,000 bicloride solution after ministering to the patient. The thermometer should be subjected to the same process.

Purification of infected water, inspection of sewerage and drains while important in the prophylaxis of typhoid fever, fall within the domain of the public health authorities rather than the private physician.

TREATMENT.

There are some members of the medical profession who take a pessimistic view of the treatment of typhoid fever, believing that we have no drugs capable of exerting a favorable influence over this disease, but that all that is required is a good nurse. On the other hand, special forms of treatment have been brought forward from time to time with extravagant claims in their behalf.

Let us not go to the extreme of optimism on the one hand, nor should we be pessimists, like Osler, who casts a shadow over therapeutic progress by the assertion that "we pour drugs of which we know little, into bodies of which we know less." But rather should be take the middle ground in our battle with this disease, using intelligently the means at our command, ever bearing in mind the limitations as well as the possibilities of medicine.

Of the utmost importance in the treatment of typhoid fever, is absolute rest in bed from the first prodromal symptoms, if we see the case so early, until convalescence is well established. It is a matter of common observation that persons stricken with typhoid fever who are compelled to make a journey or undergo other physical exertion while ill, are almost certain to develop a severe attack. To this factor, I believe is due in great measure the high mortality of typhoid fever among patients sent to hospitals from private practice.

Fresh air is as essential in the successful treatment of typhoid fever as it is in tuberculosis. Statistics show a lower rate of mortality among cases treated in tents and open shanties than in private residences and hospitals, and this difference can be due to no other cause than fresh air.

The diet must be fluid from the beginning of the illness until there has been no elevation of the evening temperature for at least a week. Milk is conceded by most authorities to be the best diet in typhoid, though it may be supplemented with animal broths or raw egg albumen in water especially in those patients who find difficulty in digesting milk, vomiting it or passing it in curds in the stools. Boiling the milk for a few days or giving a dose of lime water or a little pepsin with it will often aid in its digestion in these cases.

Hydrotherapy as advocated by Brand, with its modifications, also the internal use of antiseptics are methods of treatment to which have been ascribed specific virtue. While they cannot be regarded as specifics there is no doubt of their great value as adjuncts in the general management of this disease.

The length of this paper will not permit a detailed description of the Brand treatment by means of cold baths, with which we are familiar, and which, by the way, is utterly impracticable in private practice in the country where we are often denied the luxury of a wash-nay and a bath-tug is a thing unknown. We can get the good effects of the cold bath, such as reduction of temperature, diminished frequency and increased force of the pulse, sedation of the nervous system and induction of sleep, by means of tepid sponging with brisk friction, without the shock and work and exertion of the patient incident to the dub bath or cold pack.

Many drugs have been used in the treatment of typhoid fever with varying degrees of success. My individual experience convinces me that those of greatest benefit are quinine, calomel, turpentine, alcohol and strychnia, and the mineral acids, my preference of which is the aromatic sulphuric.

So firmly convinced am I of the efficiency of quinine, that I would be loath to take charge of a case of typhoid fever without this drug; and were I restricted to the use of one remedy, quinine would be my choice. One has but to observe the effect of withdrawal of this remedy in a case doing well—watch the temperature go up, the tongue become brown and cracked, the abdomen tympanitic and nervous symptoms develop, all going to show that the bacilli infesting the intestinal tract are rapidly elaborating toxins which being absorbed into the blood are overpowering the vital powers of the patient—to be convinced of the value of quinine.

I do not indorse the enormous doses advocated by Liebermeister, nor do I believe that it should be given at all for its *antipyretic* effect, but it should be given throughout the course of the disease in dosage sufficient to keep the system under its influence and maintain its antidotal effect upon the toxins that are being absorbed into the blood.

If quinine can kill the malarial parasite in the blood, which it does in so weak a dilution as 1 to 20,000, why would it not have some neutralizing effect upon toxins elaborated by other forms of bacteria?

Too much stress cannot be placed upon the inportance of elimination. All of the organs should be kept active. The bowels to carry off the rapidly multiplying bacteria and fermenting refuse of food; the skin, kidneys and lymphatic glands to deal with the toxins which have been already absorbed, and the liver to pour out nature's antiseptic, the bile, which passing down the intestinal tract exerts its antifermentative and bacteriolytic influence throughout its course. All of these indications are met by calomel. Being a laxative, an efficient diuretic, and having a selective action upon the liver and excrementitious intestinal glands, it carries along smoothly the process of elimination, and the result of *faulty* elimination—tympanites, weak heart action, dry, brown and cracked tongue, prostration and nervous irritation as shown by subsultus tendinum, carphologia and other evidences of the typhoid state are held in abeyance.

At least one or two free bowel movements should be had in twenty-four hours. If the bowels become too active, the astringent effect of aromatic sulphuric acid, aided if necessary by bismuth of sulphorcarboliate of zinc will hold them within bounds, so that we have to contend with neither constipation nor diarrhoea.

At the beginning of the illness a good calomel purge with podophylin should be given until the bowels are thoroughly emptied, the calomel should be continued in dosage of one to two grains a day supplemented by other laxatives as occasion demands. Should tympanites develop, turpentine internally and locally is the best remedy.

I do not approve of the promiscuous use of stimulants, but if in spite of our best efforts the case sinks into the typhoid state, stimulants are demanded, and of these alcohol and strychnine hold the first rank. Their use should not be postponed until symptoms of adguamia are well developed, but should be given upon the first signs of warning—dry tongue, irregular, intermittent or dicrotic pulse, and indistinct first sound of the heart.

Intestinal hemorrhage should be treated by cold to the abdomen, absolute rest and the administration of adrenalin and acetate of lead and opium with the subcutaneous use of normal salt solution if the hemorrhage has been profuse.

If perforation occur, the only hope lies in surgery, and Keen says the operation is hopeless unless done within twenty-four hours.

A seemingly trivial matter, but really of importance, is attention to the mouth during the course of the disease. If sordes is allowed to accumulate and decompose upon the teeth, it furnishes a favorable nidus for the growth of various bacteria which may enter the system through the tonsils or be swallowed or inspired and give rise to grave complications. A saturated solution of barocic acid, or one of the analogues of listerine makes an excellent wash for cleansing the mouth and teeth.

The management of convalescence requires strict supervision of the diet. No change in the diet should be allowed until the third or fourth day after the temperature has returned to normal, when a soft boiled egg or a little milk toast may be given. The condition of the pulse and appearance of the tongue should guide us as well as the temperature in forming an opinion as to the proper time to allow an increased diet and to let the patient leave the bed.

